# ^68^Ga-PSMA-11 PET/MRI: determining ideal acquisition times to reduce noise and increase image quality

**DOI:** 10.1186/s40658-020-00322-x

**Published:** 2020-08-26

**Authors:** Vishnu Murthy, Raven L. Smith, Dora H. Tao, Courtney A. Lawhn-Heath, Dave E. Korenchan, Peder E. Z. Larson, Robert R. Flavell, Thomas A. Hope

**Affiliations:** 1 Department of Radiology and Biomedical Imaging, University of California San Francisco, San Francisco, CA USA; 2 UCSF Helen Diller Family Comprehensive Cancer Center, University of California San Francisco, San Francisco, CA USA; 3 Department of Radiology, San Francisco VA Medical Center, San Francisco, CA USA

## Abstract

**Background:**

In this study, we investigate the impact of increased PET acquisition time per bed position on lesion detectability, standard uptake value, and image noise in ^68^Ga-PSMA-11 PET/MRI scans.

**Methods:**

Scans of twenty patients were analyzed in this study. Patients were injected with ^68^Ga-PSMA-11 (mean, 5.50 ± 1.49 mCi) and imaged on a 3.0 T time-of-flight PET/MRI. PET images were retrospectively reconstructed using 0.5, 1, 2, 4, 7, and 10 min of PET data. Lesion detectability was evaluated on a 5-point Likert Scale for each lesion in each reconstruction. Quantitative analysis was performed measuring image noise and lesion uptake.

**Results:**

A total of 55 lesions were identified, and lesion detectability increased from 2.07 ± 1.14 for 0.5 min to 4.93 ± 0.26 for 10 min (*p* < 0.001), with no significant difference detected between 7 and 10 min of scan time. Average SUV_max_ decreased from 9.89 ± 6.62 for 0.5 min to 8.64 ± 6.81 for 10 min. Noise decreased from 0.72 ± 0.22 for 0.5 min to 0.31 ± 0.12 for 10 min (*p* < 0.001) and were nearly equivalent between 7 and 10 min. Pairwise interaction terms between size, SUV_max_, and scan time were all found to be significant, although the interaction term between SUV_max_ and scan time was found to be the most significant.

**Conclusions:**

Increased acquisition duration improves image quality by increasing detectability and reducing noise. In patients with biochemical recurrence, increased acquisition time up to 7 min improves lesion detection.

## Introduction

Prostate cancer is one of the most prevalent types of cancer found in men [1]. Prostate-specific membrane antigen (PSMA) is a transmembrane protein that is overexpressed in nearly all prostate cancers and remains a useful diagnostic and therapeutic target [2–4]. Radiotracers targeting PSMA have shown higher sensitivity compared to conventional imaging for the detection of recurrent prostate cancer [5–7]. Due to its high detection rate for localization of recurrent prostate cancer, ^68^Ga-PSMA-11 is increasingly being used to assess biochemical recurrence in prostate cancer patients after prostatectomy or radiation therapy [5, 8].

Compared with PET/CT, PET/MRI promises further advances in multimodal imaging of prostate cancer by providing better soft tissue contrast, reduced radiation exposure, as well as additional parameters such as diffusion and perfusion [9]. Several studies have also demonstrated a high overall sensitivity and specificity of multi-parametric MRI using T2-weighted imaging, diffusion-weighted MRI, and dynamic contrast-enhanced MRI in prostate cancer detection [10]. In one study, recurrent prostate cancer was also detected more easily and more accurately with ^68^Ga-PSMA-11 PET/MRI than with PET/CT [11]. In PET/MRI workflow, MRI images are acquired simultaneously with PET data, and as MRI images frequently take longer to acquire than standard PET beds, longer PET acquisitions are possible [12]. In rectal cancer, prolonged PET acquisitions have been shown to increase the detection of local regional nodes [13]. Additionally, increased acquisition time per bed position has been thought to improve image quality and reduce image noise. Using non-time-of flight PET/MRI, Lütje et al. found that lesion detectability linearly increased with increasing acquisition time for a single bed position (pelvis) [14]. Similarly, Noto et al. found that reduction of acquisition duration results in an increase in likelihood of halo artefacts, especially with durations lower than 180s per bed position, using whole-body acquisitions [15].

It is unclear how longer acquisition times impacts PET/MRI detection sensitivity in whole-body PET acquisitions, and furthermore, it is unclear how increased acquisition time impacts detectability with time-of-flight scanners. Therefore, the objective of this study is to determine the impact of PET acquisition duration on image noise, standard uptake values (SUV), and lesion detectability using a time-of-flight simultaneous PET/MRI system.

## Materials and methods

^68^Ga-PSMA-11 PET/MRI scans of twenty patients with biochemically recurrent prostate cancer were analyzed in this study. Patients were imaged as part of an existing ^68^Ga-PSMA-11 study (NCT02918357), and the analysis was done retrospectively. This trial was performed under an Investigational New Drug Approval from the Food and Drug Administration. Additionally, this study was approved by the institutional review board, and written informed consent was obtained from all patients prior to any study procedures.

### ^68^Ga-PSMA-11 PET/MRI acquisition and reconstruction

Patients were injected with ^68^Ga-PSMA-11 (mean, 5.50 ± 1.49 mCi) and imaged 64.9 ± 9.7 min after injection on a 3.0 T time-of-flight PET/MRI (GE Healthcare, Waukesha, WI, USA). PET/MRI images were acquired in list mode, and a 10-min acquisition time per bed position was used for the abdomen and pelvis bed positions. In addition to the 10-min PET acquisition obtained at the abdomen and pelvis bed positions, a PET acquisition was obtained from the upper abdomen to the vertex for 3 min per bed position.

Attenuation-corrected PET images from the abdomen and pelvis were retrospectively reconstructed using PET data from the original 10-min acquisition. Each reconstruction consisted of PET data from the start of the 10-min acquisition to the specified time point (0.5, 1, 2, 4, 7, or 10 min). The acquired PET images were reconstructed using time-of-flight Ordered Subset Expectation Maximization (OSEM) using 2 iterations and 28 subsets and a matrix size of 256 × 256, with a 600 × 250-mm field of view and a slice thickness of 2.8 mm. MRI sequences were previously described [12], and ^68^Ga-PSMA-11 was synthesized as previously described [16].

### Qualitative and quantitative image analysis

A nuclear medicine physician (TH) rated each individual lesion for each reconstruction for image quality using a five-point Likert scale (1—not visible, 5—clearly visible above the noise). Reference region of interests (ROIs) were manually positioned to include lesions and the muscle-to-measure noise. Noise was measured as the standard deviation of the muscle divided by the SUV_mean_ of the muscle [17].

### Statistical plan

Likert score distributions at each scan time were compared using least square means for multiple comparisons with a Tukey correction, using a repeated measures design. Likert score was also modeled as a function of size, SUV_max_, and scan time, treating lesions as a blocking variable. The influence of scan time, SUV_max_, and lesion size on Likert score was investigated via repeated ordinal regression. A cumulative linked mixed model was specified for Likert score as the dependent variable and including interaction terms between the three independent variables. Separate lesions were treated as random variables. The influence of scan time on SUV_max_ was determined by constructing a linear mixed-effects model of SUV_max_ as a function of scan time, treating the individual lesions as a random variable. For each lesion, the SUV_max_ at each time point was normalized by the SUV_max_ at 10 min in order to represent the percentage change in SUV_max_ over time. The strength of the interaction was quantified using a Spearman correlation between normalized SUV_max_ and scan time. Statistical analyses were performed using R open-source statistical software (https://www.r-project.org/). For all statistical models constructed, an analysis of variance (type II) was performed in order to determine statistical significance of each term in the model not being equal to zero. A *p* value of 0.05 or less was considered to be statistically significant.

## Results

### Qualitative evaluation

**Fig. 1:**
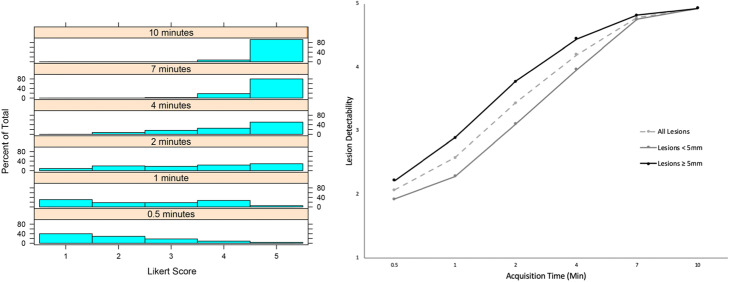
Lesion detectability (mean Likert Score) increased as acquisition time increased (left, *p* < .001), and there was no significant difference between 7 and 10 min of acquisition time. For acquisition times less than 10 min per bed position, lesions ≥ 5 mm were more detectable compared with lesions < 5 mm in size (right). This graph demonstrates that small lesions need longer acquisition times to have the same detectability as large lesions (*p* = .036)

**Fig. 2:**
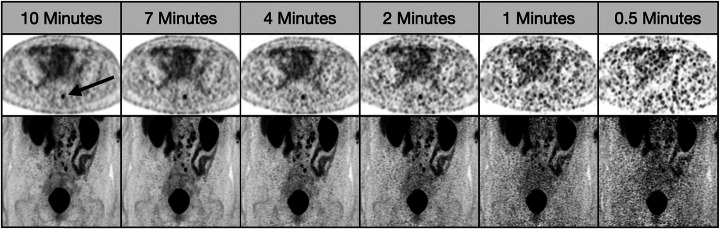
Figure illustrating how lesion detectability increases as acquisition time per bed position increases in axial slices and coronal MIP (maximal intensity projection) images. In this patient, a perirectal node (black arrow) is clearly visualizable in the 10- and 7-min images, but is not distinguishable at the 1-min or 0.5-min reconstructions due to the increased noise of the images

A total of 55 lesions were included in the 20 patients evaluated. As acquisition time increased, qualitative lesion characterization improved with qualitative analysis of PET images ranging from 2.07 ± 1.14 for 0.5 min, 2.58 ± 1.33 for 1 min, 3.44 ± 1.34 for 2 min, 4.2 ± 0.97 for 4 min, 4.78 ± 0.46 for 7 min, and 4.93 ± 0.26 for 10 min based on the Likert scale (*p* < .001) (Fig. 1). Additionally, small lesions required longer acquisition times to have the same detectability as large lesions (Fig. 1). Histograms depicting the Likert score distributions across all lesions at each scan time are also shown in Fig. 1 and demonstrate that the mean Likert score increases as a function of scan time. There was a statistically significant improvement in Likert score for each increase in acquisition time except for between 7 and 10 min. Axial slices and coronal MIP (maximal intensity projection) images for one patient are also shown in Fig. 2 and illustrate how lesion detectability increases as acquisition time per bed position increases.

Likert score was found to be most significantly affected by scan time (*p* < 0.001), confirming the trend viewed in Fig. 1. Although pairwise interaction terms between size, SUV_max_, and scan time were all statistically significant (*p* < 0.05), the interaction term between scan time and SUV_max_ was most significant (*p* < 0.001).

### Quantitative evaluation

**Fig. 3:**
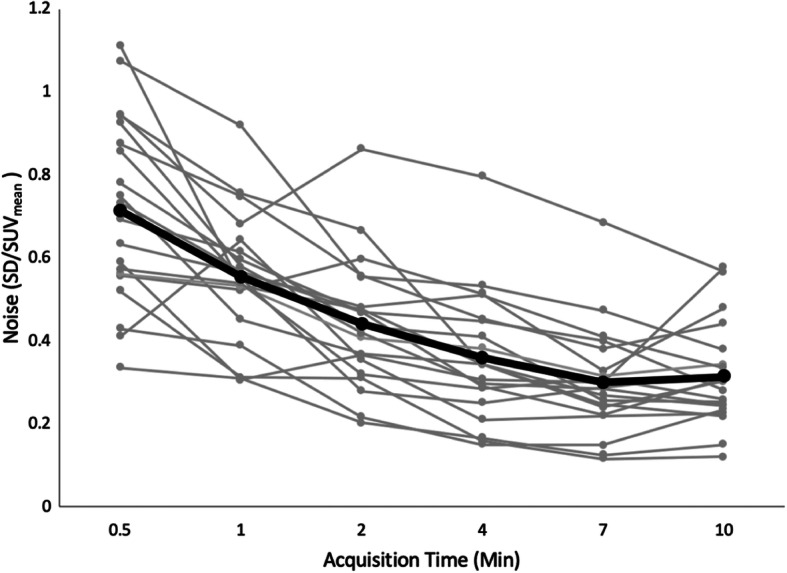
Graph illustrating how noise (SD/SUV_mean_) changed as acquisition time per bed position increased for all twenty patients. As acquisition time increased, average noise levels (represented by the black line) decreased from 0.72 ± 0.22 for 0.5 min to 0.31 ± 0.12 for 10 min and were nearly equivalent between 7 and 10 min of acquisition time

Quantitatively, as acquisition time increased, average noise levels decreased from 0.72 ± 0.22 for 0.5 min to 0.31 ± 0.12 for 10 min and were nearly equivalent between 7 and 10 min of acquisition time (Fig. 3). All reconstructions greater than 1 min per bed had an average noise less than 0.5 (Fig. 3).

**Fig. 4:**
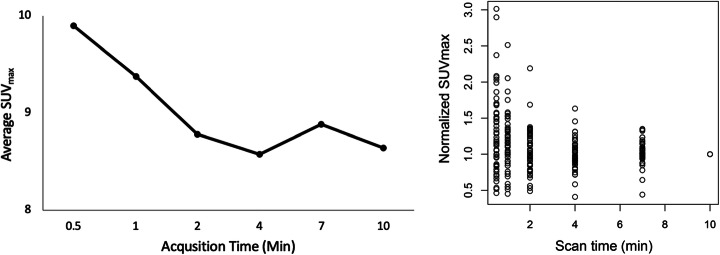
As acquisition time increased, average SUV_max_ decreased from 9.89 ± 6.62 for 0.5 min to 8.64 ± 6.81 for 10 min and was lowest at an acquisition time of 4 min per bed position (left). On average, the SUV_max_ normalized to the 10 min value decreased from 1.05 ± 0.29 at 2 min to 1.00 at 10 min of scan time (right, *p* = .001)

The average SUV_max_ of the lesions decreased as the acquisition time increased (9.89 ± 6.62 for 0.5 min to 8.64 ± 6.81 for 10 min) and was lowest (8.58 ± 6.61) at an acquisition time of 4 min (Fig. 4). The SUV_max_ normalized to the 10-min value was found to be significantly affected by scan time (*p* = 0.001) with a weakly negative trend (*ρ* = − 0.18) and, on average, decreased from 1.05 ± 0.29 at 2 min to 1.00 at 10 min of scan time (Fig. 4).

## Discussion

**Fig. 5:**
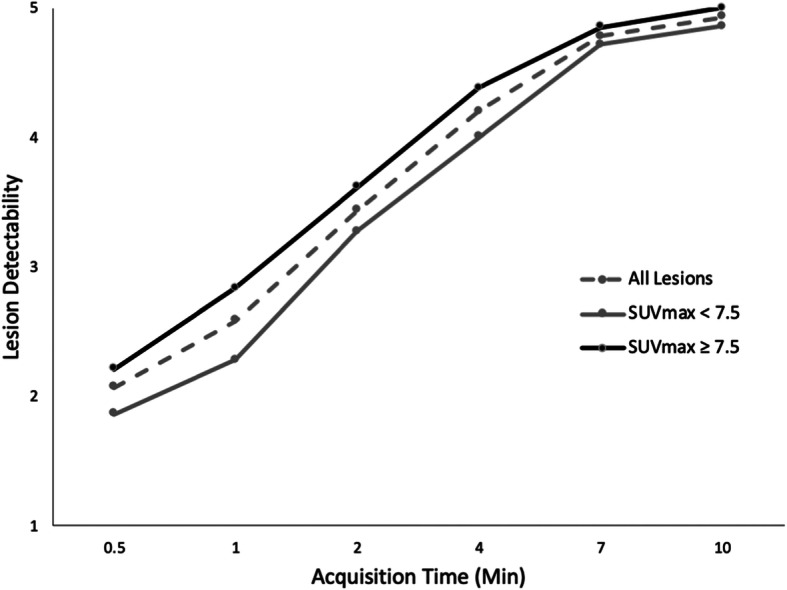
Lesion detectability increased as acquisition time increased, and for all acquisition times, lesions with SUVmax ≥ 7.5 were more detectable compared with lesions with SUVmax < 7.5, though this difference narrows at longer acquisition times. This graph demonstrates that lesions with mild uptake need longer acquisition times to have similar detectability as lesions with intense uptake (*p* < .001)

Increased acquisition time improves overall image quality in patients by reducing image noise, which results in increased lesion detectability (Fig 5). However, no significant difference was detected between 7 and 10 min of scan time, suggesting that scanning longer than 7 min did not significantly improve the ability to identify lesions. In addition to acquisition time, qualitative lesion characterization was also significantly impacted by lesion uptake (SUV_max_), indicating that longer scan times would be more beneficial for lesions with lower uptake. In addition to acquisition time, qualitative lesion characterization was also significantly impacted by lesion size, indicating that longer scan times would be more beneficial for small lesions than for large lesions. Furthermore, the interaction term between scan time and SUV_max_ was most significant, which suggests that SUV_max_ is more important than lesion size in the ability to detect an individual lesion. SUV_max_ also generally decreased slightly across all lesions as the scan time was increased, due to the decrease in noise with increased acquisition time.

These results are similar to the results of Lütje et al., who found that lesion detectability linearly increased with increased acquisition time in ^68^Ga-PSMA-11 PET/MRI [14]. However, Lütje et al. found that lesion detectability reached a maximum at PET acquisition times of 4 min due to reduced PET signal intensity around the urinary bladder at longer acquisition times. While our results show that acquisition times longer than 7 min did not significantly alter lesion detectability, it is important to note that Lütje et al. imaged one bed position (pelvis) on a non-time-of-flight scanner and had an increased uptake time of ^68^Ga-PSMA-11 (mean of 168 min, range of 77–320 min). Using whole-body ^68^Ga-PSMA-11 PET/MRI acquisitions, Noto et al. also found that acquisition times less than 3 min per bed position lead to the introduction of unacceptable image artefacts and decreased diagnostic performance [15]. Furthermore, Noto et al. also imaged patients approximately 1 h postinjection, but used a non-time-of-flight scanner. Our results build on the findings of Noto et al. by exploring the effect of increased acquisition times on image quality using a time-of-flight PET/MRI scanner. Neither article looked at acquisition times longer than 5 min.

It also has to be noted that while acquisition times were varied, the uptake time after injection was kept fairly consistent across all patients analyzed in this study and was in accordance with EANM/SNMMI guidelines [18]. However, recent research has also demonstrated the potential benefits of delayed imaging protocols. Hohberg et al. demonstrate that using ^68^Ga-PSMA-11, there was a higher lesion detection rate at a later imaging time point (3 h postinjection vs. 1 h postinjection) when imaging patients with recurrent prostate cancer with low PSA levels [19]. When imaging patients with biochemically recurrent prostate cancer using ^68^Ga-PSMA I&T, Schmuck et al. also demonstrated that the tumor-to-background ratio in the prostate gland improved over time [20]. Therefore, while delayed imaging was not explored in this study, it would also likely improve uptake and lesion detectability.

In addition to ^68^Ga-labelled PSMA radiotracers, 18F-labelled PSMA imaging agents have also become more numerous and can offer potential benefits over ^68^Ga-labelled tracers as well. Unlike ^68^Ga agents, 18F agents have a higher potential for delivery to smaller hospitals and PET centers [21]. Furthermore, cyclotron-based synthesis of 18F imaging agents allows for synthesis of higher activity, and the lower positron energies of 18F may result in sharper imaging [21]. It is possible that in the near future, cyclotron production of ^68^Ga may allow for increased administered doses of ^68^Ga-PSMA-11.

Overall, it should be noted that acquisition parameters can have a significant impact on the ability of PSMA PET-targeted radiotracers to detect metastatic prostate cancer. When comparing radiopharmaceuticals, it is important to take this into consideration. By changing scanner type, acquisition time, imaging delay, or injected activity, the detection sensitivity of an individual radiotracer can be altered dramatically. In some literature, head-to-head comparisons are performed with dramatically different acquisition parameters, which can bias the results toward a specific radiotracer [22]. In general, when comparing PSMA radiotracers, technical differences in acquisitions have a greater impact on patient-level sensitivity than the differences in individual radiopharmaceuticals.

This study has several limitations. Delays in imaging from clinical workflow could potentially cause differences in lesion detectability. An additional limitation of this study is the single-center acquisition on one scanner type and a relatively small number of patients. In addition, further research is required to determine whether higher doses of ^68^Ga-PSMA-11 can result in improved lesion detectability and image quality as well.

## Conclusion

As ^68^Ga-PSMA-11 becomes more widely used in imaging prostate cancer, determining ideal acquisition times in PET/MRI has become an important challenge. We find that increased acquisition duration improves image quality and reduces noise in ^68^Ga-PSMA-11 PET/MRI scans. An acquisition time of 7 min per bed position results in the lowest noise, and scanning longer than 7 min per bed position did not significantly increase lesion detectability. Further research is necessary to explore how these results may be replicated using other PSMA-targeting imaging agents.

## Data Availability

The datasets used and/or analyzed during the current study are available from the corresponding author on reasonable request.
